# A Numerical Implementation to Calculate Elastic Properties of Biological Membrane Simulations

**DOI:** 10.1021/acs.jcim.6c00143

**Published:** 2026-07-09

**Authors:** Denys E. S. Santos, Vinicius Firmino, Ricardo L. Longo, Thereza A. Soares

**Affiliations:** † Department of Fundamental Chemistry, 28116 Federal University of Pernambuco, Cidade Universitária, 50740-560 Recife, Brazil; ‡ Department of Chemistry, FFCLRP, 28133 University of São Paulo, 14040-901 Ribeirão Preto, Brazil; § Hylleraas Centre for Quantum Molecular Sciences, University of Oslo, 0315 Oslo, Norway

## Abstract

The elastic properties of biological membranes can be described by mechanical constants like the bending modulus at flexure (*k*_c_) and the area compressibility modulus (*K*_A_), which quantify the energy cost associated with bending, compression, and stretching of the membrane area. These properties provide a means to describe phenomena such as the shape variation of a vesicle in response to pressure or the strain energy of a membrane influenced by lipid composition, interactions with ions, small molecules, or biomolecules. The determination of elastic moduli provides a quantitative basis for describing deformation-related processes in cellular systems at the molecular and mesoscopic levels. However, measurements of elastic constants, such as the bending modulus, exhibit a wide dispersion of reported values, ranging from approximately 10 *k*_B_ *T* (4 × 10^–20^ J) to 100 *k*_B_ *T* (4 × 10^–19^ J) for liquid disordered phospholipid membranes. Computational protocols for estimating elastic constants of lipid membranes commonly rely on Fourier analysis of the membrane surface, where the upper integration limit is determined by lipid molecular dimensions, making results sensitive to lipid composition and complicating cross-system comparisons. We have implemented the *s_comp* tool in the SuAVE software to estimate area compressibility from the direct integration of membrane surface areas, circumventing the dependence on wavevector integration limits that arises when elastic constants are extracted from Fourier mode amplitude spectra. The calculation does not require prior knowledge of lipid molecular dimensions and is therefore applicable to membranes of arbitrary composition and morphology. The calculated elastic constants are in reasonable agreement with values reported in the literature, falling within the variability observed across computational protocols and experimental techniques. As with any fluctuation-derived property, fully converged trajectories and appropriate statistical sampling are prerequisites for reliable estimates. The SuAVE software is freely available from https://github.com/SuAVE-Software.

## Graphical abstract

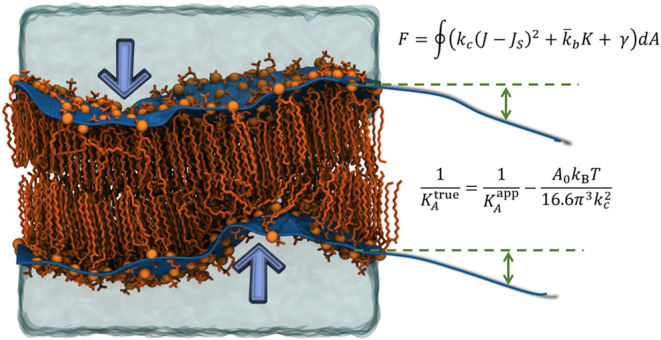

## Introduction

Biological membranes are complex structures defining the boundaries of every known cell. These structures not only serve as biomatrices for cell communication and numerous biochemical reactions crucial for sustaining life but protect and shape the cell and cellular organelles^1,2^ Deformation-dependent cellular processes require biological membranes to stretch, bend and compress while maintaining their structural integrity.^1,2^ The work required to deform a flat membrane into a variety of shapes is controlled by the membrane spontaneous curvature (*i.e*. the nondeformed, relaxed state of the membrane) and elasticity.^3^ The influential work of Helfrich^4^ established the mathematical framework to calculate the membrane bending free energy from three primary elastic modulus: the bending rigidity (*k*_c_), the Gaussian curvature modulus (*k̅*_b_) and the area compressibility modulus (*K_A_*) (for a review see refs 1 and 3)­

1 $$F=\oint ({k_{c}\left(J-J_{S}\right)}^{2}+{\mathrm{k̅}}_{b}K+\gamma )dA$$

2 $$\frac{1}{K_{A}}\equiv \frac{1}{A}{\left(\frac{dA}{d\gamma }\right)}_{V,T}$$

In eq 1, the membrane surface area is given by *A*, the local shape of the bilayer is determined by the total curvature *J*, usually taken as the mid surface between the two leaflets, and the Gaussian curvature *K*. The bilayer spontaneous curvature is defined by *J*_*S*_, which depends on the spontaneous curvatures of the inner and outer leaflets,^1,3^ and results from the asymmetric distribution of inhomogeneities of the bilayer structure throughout the bilayer thickness.^1^ The terms *k*_c_ and *k̅*_b_ represent the bending modulus and the modulus of the Gaussian curvature, respectively. In eq 2, *K_A_* is the area compressibility modulus, γ is the surface tension, and *A* is the membrane surface area represented as well in eq 1. Hence, the Helfrich Hamiltonian connects the energy cost to deform the membrane from its spontaneous (flat) shape to elastic properties that are intrinsic to the membrane composition. For this reason, it provides a robust foundation to integrate the length scales of molecular simulations and cell membrane deformation processes. There are, however, a few caveats.

The bending modulus or bending rigidity (*k*_c_) describes the resistance of a material against bending deformations, being a crucial parameter for assessing how soft materials respond to mechanical stresses, change shapes and structures. *k*_c_ is an intrinsic property of the membrane and thus dependent on lipid composition, such as the length or the degree of unsaturation of the aliphatic chains. A number of techniques have been developed to measure *k*_c_,^5–9^ which varies from 10 *k*_B_ *T* (4 × 10^–20^ J) to 100 *k*_B_ *T* (4 × 10^–19^ J) for liquid disordered phospholipids. The Gaussian curvature modulus (*k̅*_b_) describes the elastic energy accumulated within the bilayer in response to the generation of the Gaussian curvature *K*. In contrast to bending modulus, the elastic constant *k̅*_b_ is notoriously difficult to measure,^1,2^ once most experimental techniques used to characterize membrane elasticity (X-ray, pipet aspiration and neutron spin echo experiments) primarily probe mean curvature deformations, which are governed by the bending modulus *k*_c_.^5–9^ These methods do not induce or isolate Gaussian curvature changes and therefore provide little to no direct sensitivity to *k̅*_b_. However, recent advances have been reported from computational simulations,^10–12^ allowing for the estimation of this elastic constant. The area compressibility modulus or area elastic modulus describes the resistance of the membrane to changes in its surface, *i.e*. the energy cost per unit area to change the surface area of a membrane. Experimental results of *K_A_* are derived from the expansion of the bilayer area in response to changes in surface tension using various techniques.^9^ Values of *K_A_* vary depending on the type of phospholipid, fatty acid composition, the presence of cholesterol, environmental conditions (*e.g*. temperature and pH) and the technique used for the measurements. Furthermore, these measurements are limited to a small number of lipids and even fewer lipid mixtures.^6^

Therefore, there is a sizable potential for the development of computational tools to enable the calculation of elastic modulus from molecular simulations of complex lipid mixtures. Computational methods such as real-space fluctuation analyses^13–15^ and correlation-based approaches^16^ have emerged as efficient alternatives for estimating elastic properties. Real-space fluctuation methods typically rely on the discrete sampling of atomic positions, making the results dependent on the spatial resolution and the specific definition of the membrane surface. In contrast, correlation-based approaches are often formulated in terms of density correlation functions, which commonly assume planar or quasi-planar interfacial profiles for their evaluation. As a consequence, real-space methods may be sensitive to the discretization scheme used to represent the surface, while correlation-based approaches may be limited by the assumption of planar density distributions. These limitations become particularly relevant when dealing with curved interfaces or systems exhibiting complex morphological features.

Despite the considerable scientific efforts invested in the development of mathematical models^7,17,18^ and the rapid advancement of computational methods for biomolecular simulations, there remains a need for robust computational tools to extracting comprehensive mechanical information from molecular simulations of soft matter.^6,7,17,19^ Data analysis is fundamental for the construction and development of predictive models, and the success of these models is directly linked to the accuracy and efficiency of the analyses in measuring relevant properties. In this work, we report on the implementation of a numerical approach, derived from the theoretical formulation proposed by Waheed and Edholm,^17^ to calculate the elastic constants *k*_c_ and *K_A_* from molecular simulations of soft materials within the SuAVE framework. This approach enables the evaluation of elastic properties without relying on assumptions regarding molecular length scales, Fourier modes, specific levels of coarse-graining, or, importantly, membrane morphology. We have compared the calculated values against a standardized data set of lipid membranes^6,18–21^ for which the elastic constants have been previously obtained by computational and experimental means.

## Theoretical Background

The area compressibility, *K_A_*, embodies a confluence of various factors encompassing both undulatory and peristaltic motions within a lipid membrane.^18^ These factors, in turn, cause the experimentally measured compressibility to present contributions from the intrinsic and undulatory membrane motions. The intrinsic compressibility, *K*_*A*_^true^, is related to the change in the true local area of a resting bilayer, whereas the undulatory compressibility, *K*_*A*_^und^, brings contributions to the changes in the projected area caused by membrane undulations at the constant true local area. Assuming that these contributions are independent from each other, it is possible to describe their combination as follows.^17,22^

3 $$\frac{1}{K_{A}^{\mathrm{app}}}=\frac{1}{K_{A}^{\mathrm{true}}}+\frac{1}{K_{A}^{\mathrm{und}}}$$

where *K*_*A*_^app^ is relative to the projected area of the membrane, while the undulatory contributions are added by the term *K*_*A*_^und^, resulting in the contribution of the oscillation of the real membrane area, *K*_*A*_^true^. Each of these terms can be obtained by applying the ensemble theory to eq 2 to obtain eq 4

4 $$\frac{1}{K_{A}}=\frac{\sigma_{A}^{2}}{Ak_{B}T}=\frac{\sigma_{a}^{2}N}{2ak_{B}T}$$

where *a* = 2*A*/*N* is the area per lipid, *N* the number of lipids constituting the bilayer, σ_*a*_^2^ = σ_*A*_^2^(2*N*)^2^ is the mean-square fluctuation of the area per lipid, *k*_B_ the constant of Boltzmann and *T* the temperature.

Notwithstanding the relation between the intrinsic and undulatory membrane compressibilities (eq 3), the assessment of these constants requires accurate values for the real (curved) and projected area to be estimated. This task can be achieved through the use of the Fourier expansion of the surface function (eq 5). This is especially important for surfaces containing ripples or curvatures resulting from thermal fluctuations as is often the case of biological membranes.^3,17,18,22^

5 $$A=A_{0}\left(1+\frac{1}{2}\sum_{q}q^{2}{\left|u_{q}\right|}^{2}\right)$$

where *A* is the real area, *A*_0_ is the projected area, *u*_*q*_ is the amplitude of the Fourier modes and **q** is the bidimensional wave vector. The application of Helfrich’s Hamiltonian (eq 1) provides the values of |*u*_*q*_| in terms of the mechanical properties of lipid membranes (eq 6).

6 $$E=\frac{A_{0}}{2}\sum_{q}{\left|u_{q}\right|}^{2}\left(k_{c}q^{4}+\gamma q^{2}\right)$$

Through the use of the Equipartition Theory, eq 6 may be rewritten as stated in eq 7, that in combination with eq 5 gives rise to eq 8 as a way for the assessment of area fluctuation in terms of mechanical properties of the lipid bilayers. For more details see refs 3, 17, 18, and 22

7 $$\left\langle {\left|u_{q}\right|}^{2}\right\rangle =\frac{k_{B}T}{A_{0}}\frac{1}{\left({k_{c}q}^{4}+{\gamma q}^{2}\right)}$$

8 $$A=A_{0}\left(1+\frac{1}{2}\sum_{q}q^{2}\frac{k_{b}T}{A_{0}}\frac{1}{\left({k_{c}q}^{4}+{\gamma q}^{2}\right)}\right)=A_{0}+\frac{k_{b}T}{2}\sum_{q}\frac{1}{\left(k_{c}q^{2}+\gamma \right)}$$

Once this relationship is established, the area compressibilities can be derived by applying eqs 2 to 8. After straightforward algebraic simplifications, this procedure leads to eq 9. It should be noted that the wave vectors are constrained by the periodic boundary conditions, such that $q=2\pi /\sqrt{A_{0}}\left(m,n\right)$ .

9 $$\frac{1}{K_{A}^{\mathrm{true}}}=\frac{1}{K_{A}^{\mathrm{app}}}-\frac{{A_{0}}^{2}k_{B}T}{32\pi^{4}Ak_{c}^{2}}\left(1-\frac{\gamma }{K_{A}^{\mathrm{app}}}\right)\sum_{n,m}\frac{1}{{\left(n^{2}+m^{2}+A_{0}\gamma /4\pi^{2}k_{c}\right)}^{2}}$$

From eq 9, two major approaches may be carried out. The first one applies infinite upper limit to the summation and a lower cutoff at *n* ^2^ + *m* ^2^ = 1, leading to eq 10, described by Waheed and Edholm,^17^ and den Otter.^23^ The second approach is carried out with numerical integration of eq 9 with γ = 0 leading to eq 11, as described by Waheed and Edholm.

10 $$\frac{1}{K_{A}^{\mathrm{true}}}=\frac{1}{K_{A}^{\mathrm{app}}}-\frac{A_{0}k_{B}T}{32\pi^{3}k_{c}^{2}}$$

11 $$\frac{1}{K_{A}^{\mathrm{true}}}=\frac{1}{K_{A}^{\mathrm{app}}}-\frac{A_{0}k_{B}T}{16.6\pi^{3}k_{c}^{2}}$$

Both expressions, obtained by Waheed and Edholm,^17^ and den Otter,^23^ can be applied to systems where γ = 0, being an appropriate approximation for simulations of soft matter under NPT conditions. However, the correct assessment of the area compressibility constants is essential to obtain an estimate of the bending modulus of lipid membranes.

Despite providing the formulation in eq 5, determining surface areas and their corresponding area compressibility moduli requires the integration boundaries from eq 8. Furthermore, the definition of these integration limits may vary depending on the lipid composition. Specifically, the upper limit is contingent upon the molecular size of the lipids constituting the membrane.^17^ To address this limitation, we employed the area integration procedure implemented in the *s_area* tool within SuAVE, ensuring an accurate calculation of the area compressibility by *s_comp* tool. The use of the SuAVE framework is particularly advantageous, as it allows the chemical surface to be defined using atoms, coarse-grained beads, or chemical groups. Once this definition is established, the method performs a two-dimensional fitting procedure to generate a high-resolution representation of the chemical interface. The resulting surface captures membrane undulations, morphological variations, and thermal fluctuations, thereby enabling a robust characterization of the mechanical properties of the interface. Furthermore, with this approach the area compressibilities may be calculated directly from eq 4, and the bending modulus from eqs 10 and 11.

Importantly, although the present validation is based on atomistic trajectories, the method is not limited to atomistic models. Because *K*_*A*_ is obtained from the area fluctuation relation in eq 4 and *k*_c_ is derived from the projected and real area compressibilities through eqs 10 and 11, the procedure depends on the statistical fluctuations of a well-defined membrane surface rather than on atomistic detail itself. Accordingly, the method can also be applied to coarse-grained simulations, provided that representative interfacial beads or bead groups are used to define the two membrane leaflets and that area fluctuations are adequately sampled.

## Models and Parameters

The simulated systems were chosen to allow the direct comparison with the experimental and computational data available in the literature. In particular, we have followed closely the systems and force-field simulated in ref 19. A total of eight membranes were built composed of 384 POPC (1-palmitoyl-2-oleoyl-*sn*-glycero-3-phosphocholine) or POPG (1-palmitoyl-2-oleoyl-*sn*-glycero-3-phosphoglycerol) or DMPC (1,2-dimyristoyl-*sn*-glycero-3-phosphocholine) or DPPC (1,2-dipalmitoyl-*sn*-glycero-3-phosphocholine) lipid molecules. Simulations were performed using two well-established atomistic force fields for lipids, namely, the GROMOS 54A7 ^24^ and the CHARMM 36 ^25^ force fields. All MD simulations were performed with GROMACS software version 2023.3 ^26^ using common integrators to ensure the data comparability and minimize algorithm biases, while force field specific methodological protocols were preserved. All simulations were performed for a total simulation time of 500 ns.

### Equilibration Setup

The protocol for building up the bilayers was separated in two stages. The initial step involves the formation of a small lipid bilayer, consisting of two layers with lipids arranged in an 8 × 4 configuration. This lipid bilayer patch was equilibrated following the protocol outlined in ref 27. The procedure included 1 ns of NVT equilibration at 200 K, followed by two stages of 25 ns of NPT simulation at 200 and 300 K, respectively. The pre-equilibrated system was replicated along the x and y axes, resulting in a lipid bilayer containing 192 lipids per layer with approximate dimensions of 10.53 by 11.05 nm. Table SI-1 describes water-to-lipid mass and molar ratios and ionic composition. The subsequent equilibration stage followed the same protocol as previously described. All the simulations comprising the equilibration step were performed by the use of leapfrog algorithm and a time step of 1 fs. Counterions were added in POPG system by replacing water molecules in the vicinity of the lipid headgroups. Initial velocities were taken from a Maxwell distribution at each specific temperature. Bond lengths within the solute and the geometry of water molecules were constrained using the LINCS algorithm.^28^ The temperatures of solute and solvent were controlled by separately coupling them to a velocity rescaling thermostat with a relaxation time of 0.5 ps.^29^ The pressure was maintained at 1 bar through the Berendsen pressure coupling algorithm with a coupling constant of 0.4 ps and an isothermal compressibility of 4.5 × 10^–5^ bar^–1^ as appropriate for water, with semi-isotropic coordinate scaling coupling.^30^

In order to ensure that the lipid membranes remain in the physically relevant liquid-crystalline phase (Lα), consistent with the experimental data, after equilibration step, simulations of POPC, POPG, and DMPC were conducted at 310 K, whereas the DPPC system was simulated at 330 K. This choice is particularly important because, as defined by eqs 10 and 11, the calculation of the bending moduli requires that *K*_*A*_^true^ > *K*_*A*_^app^, a condition that is satisfied when the systems are simulated above gel-to-liquid-crystalline (Lα) phase transition temperatures (see Figure SI-1). In addition, it is important to note that the present methodology relies on the statistical analysis of structural ensembles and is, in principle, independent of the sampling strategy employed to generate them. In this context, other approaches designed to enhance sampling efficiency, such as hydrogen mass repartitioning (HMR), may be used provided that they yield physically meaningful ensembles. However, because these techniques were neither employed nor explicitly validated in the present study, we cannot assess their quantitative impact on the calculated mechanical properties.

### Simulations with the GROMOS Force Field

Simulations performed with GROMOS force field were conducted with the use of a cutoff of 1.4 nm for electrostatic and van der Waals interaction. Long range electrostatic interactions were calculated by the use of Generalized Reaction-Field approach, with a dielectric constant of 61.^31^ As in the equilibration, bond lengths within the solute and the geometry of water molecules were constrained using the LINCS algorithm,^28^ allowing the simulations to be performed with a time step of 2 fs, representing a well-established compromise between numerical stability, energy conservation, and computational efficiency. The temperatures of solute and solvent were controlled by separately coupling them to a velocity rescaling thermostat with a relaxation time of 0.5 ps.^29^ The pressure was maintained at 1 bar through the Berendsen pressure coupling algorithm^30^ with a coupling constant of 0,4 ps, in a semi-isotropic coordinate scaling coupling, and an isothermal compressibility of 4.5 × 10^–5^ bar^–1^ as appropriate for water. Parrinello–Rahman pressure coupling algorithm^32^ was also applied in control simulations with a coupling constant of 5 ps. SPC water model was used as appropriate for simulations with the GROMOS force field.^33^ A neighbor list was updated every 10 steps and the center of mass was recentered at every 100 steps.

### Simulations with the CHARMM Force Field

Simulations employing the CHARMM force field were performed with a 1.2 nm cutoff for long-range interactions. Long-range electrostatic interactions were computed using the Particle Mesh Ewald (PME) method,^34^ with a grid spacing of 0.16 in Fourier space. The LINCS algorithm^28^ was employed to constrain bond lengths within the solute and to maintain the geometry of water molecules, allowing the simulations to be performed with a time step of 2 fs. The solute and solvent temperatures were independently regulated using a Nosé-Hoover thermostat^35,36^ with a 0.5 ps relaxation time. Pressure was kept constant at 1 bar using the Parrinello–Rahman pressure coupling algorithm^32^ with a coupling constant of 5 ps, employing semi-isotropic scaling and an isothermal compressibility of 4.5 × 10^–5^ bar^–1^, appropriate for water. The TIP3P water model^37^ was utilized, consistent with the CHARMM force field. The neighbor list was updated every 10 steps, and the center of mass of the system was recentered every 100 steps.

### Data Acquisition and Analysis

The SuAVE software^38,39^ was employed for all the analysis presented in this report. The acquired data was compared to experimental results and computational simulations outlined in refs 6, 7, 18–21, and 40–44 . For the computation of the projected 3D surface is necessary to choose the resolution of the interpolated surface, which can influence the calculated values of the geometrical properties. The resolution of the surface is only limited by the computational processing times which increase with the number of grid points. Hence, we benchmarked selected geometric properties via computations across varying resolution grids. We observe that property convergence is already achieved at a bin resolution of 100 as exemplified using the surface area of the system comprising POPC, modeled within the GROMOS force field (Figure SI-2). Henceforth, we have used a bin resolution of 100 for all calculations reported in this work.

Within the SuAVE framework, the *s_area* tool calculates the area per lipid, a parameter used in elastic property estimation, by triangulating the membrane interface. In this work, the membrane area is evaluated at the midplane, and the integration limits follow the periodic boundary conditions of the simulation box. This geometric decomposition allows the total area to be computed via Heron’s formula.^38^ The resulting area reflects the full three-dimensional structural features of the surface, including local curvature and fluctuations. This differs from the projected area, which, when obtained by 2D projection onto the *XY* plane, captures only the two-dimensional boundaries of the chemical interface. Additional details regarding the numerical procedures used to evaluate the structural properties of the lipid membranes can be found in refs 38 and 39 .

Convergence of MD simulations must be assessed separately for structural observables and fluctuation-derived mechanical properties, as these quantities impose different statistical requirements on the trajectory. Mean structural properties such as the area per lipid, bilayer thickness, and lipid volume are first-order moments of the sampled distribution and converge relatively rapidly once the system has relaxed from its initial configuration. Their convergence is monitored through the time evolution of these quantities over the production trajectory, as shown in Figure 1. The area compressibility modulus *K_A_*, however, is derived from the variance of the membrane surface area, a second-order moment, and its convergence imposes substantially more demanding sampling requirements. Variance estimators are sensitive to slow collective fluctuation modes, particularly long-wavelength undulations, which relax on time scales that may be comparable to or longer than the simulation length. Convergence of the mean area is therefore a necessary but not sufficient condition for convergence of *K_A_*.

**Figure 1:**
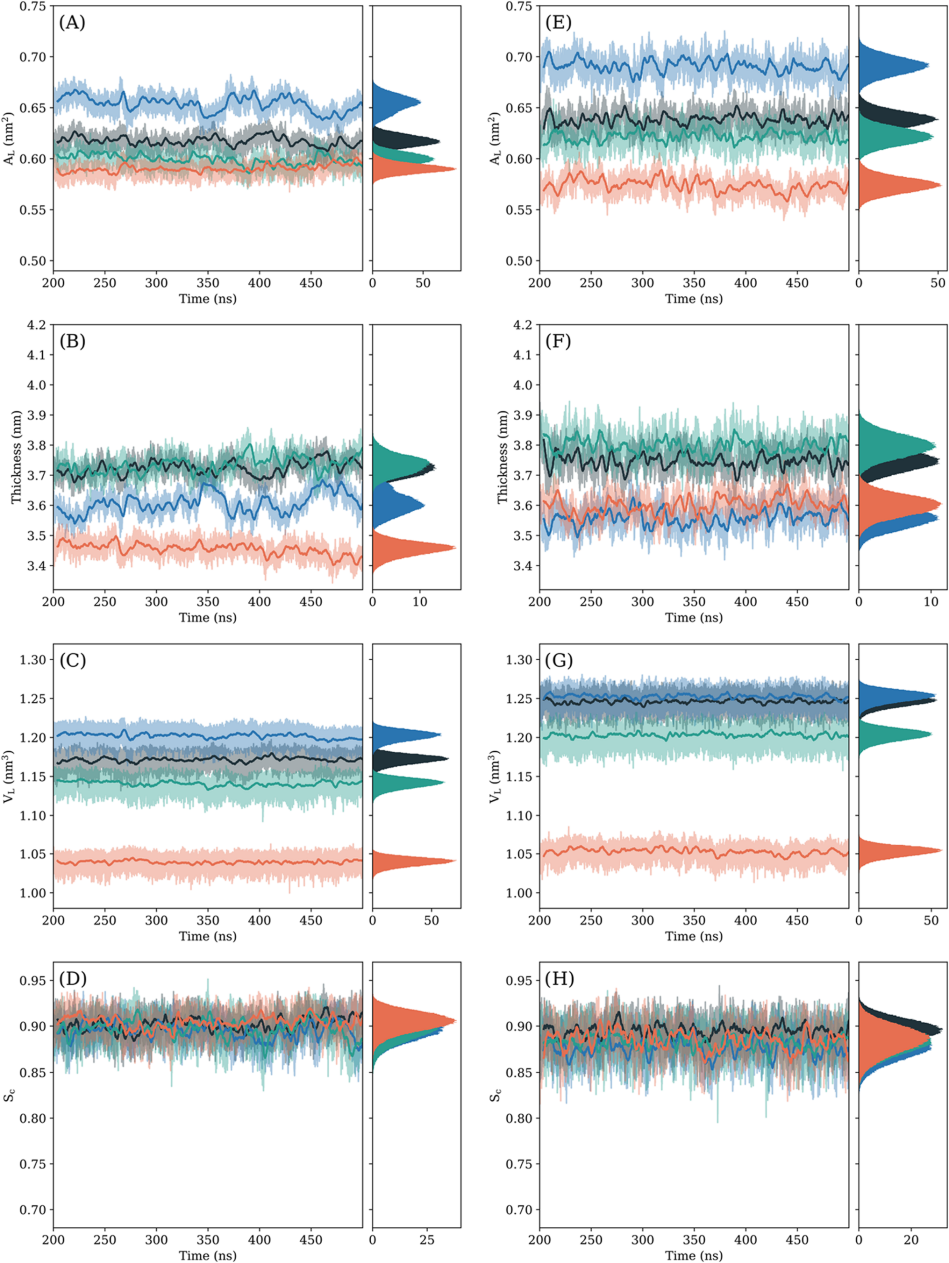
Assessment of the area per lipid (A and E), thickness (B and F), volume per lipid (C and G) and curvature order parameter (D and H), obtained with SuAVE (bin = 100), for replica 1. Trajectory averages of the last 300 ns. Simulations with GROMOS 54A7 are represented at left while simulations with CHARMM 36 at right. POPC is represented in black, while POPG in blue, DMPC in red and DPPC in green.

We have assessed the convergence of *K_A_* via two complementary strategies. First, the characteristic relaxation times of all relevant observables, including the area per lipid, bilayer thickness, lipid volume, order parameter, and *K_A_* itself, are estimated for each system and replica from the decay of their respective autocorrelation functions (Table SI-2). The longest relaxation time observed across all systems is approximately 14 ns. Second, statistical uncertainties in K_A_ are estimated using the Moving Block Bootstrap (MBB) method^45–47^ with a block size of 30 ns, approximately twice the longest observed relaxation time, ensuring that each block is statistically independent and that temporal correlations in the variance estimator are properly accounted for. Additionally, all results are averaged over three independent replicas per system (Tables SI-3 and SI-4), providing an independent assessment of the reproducibility of the fluctuation statistics across different initial conditions. These measures gauge the convergence of both first- and second-order moments of the sampled distributions and are reported in Table 1. The reported values for all structural and mechanical properties correspond to averages over three statistically independent replicas per system. For each replica, uncertainties were estimated using the Moving Block Bootstrap (MBB) procedure, with a block size of 30 ns standardized across all properties and systems, and 1000 bootstrap realizations per property.^45–47^ The final reported uncertainties combine intrareplica variance from bootstrap resampling with inter-replica variance, providing a comprehensive estimate of the total statistical error (Tables SI-3 and SI-4). The resulting distributions were further used to compute confidence intervals for all reported quantities (Table SI-5).

**Table 1:**
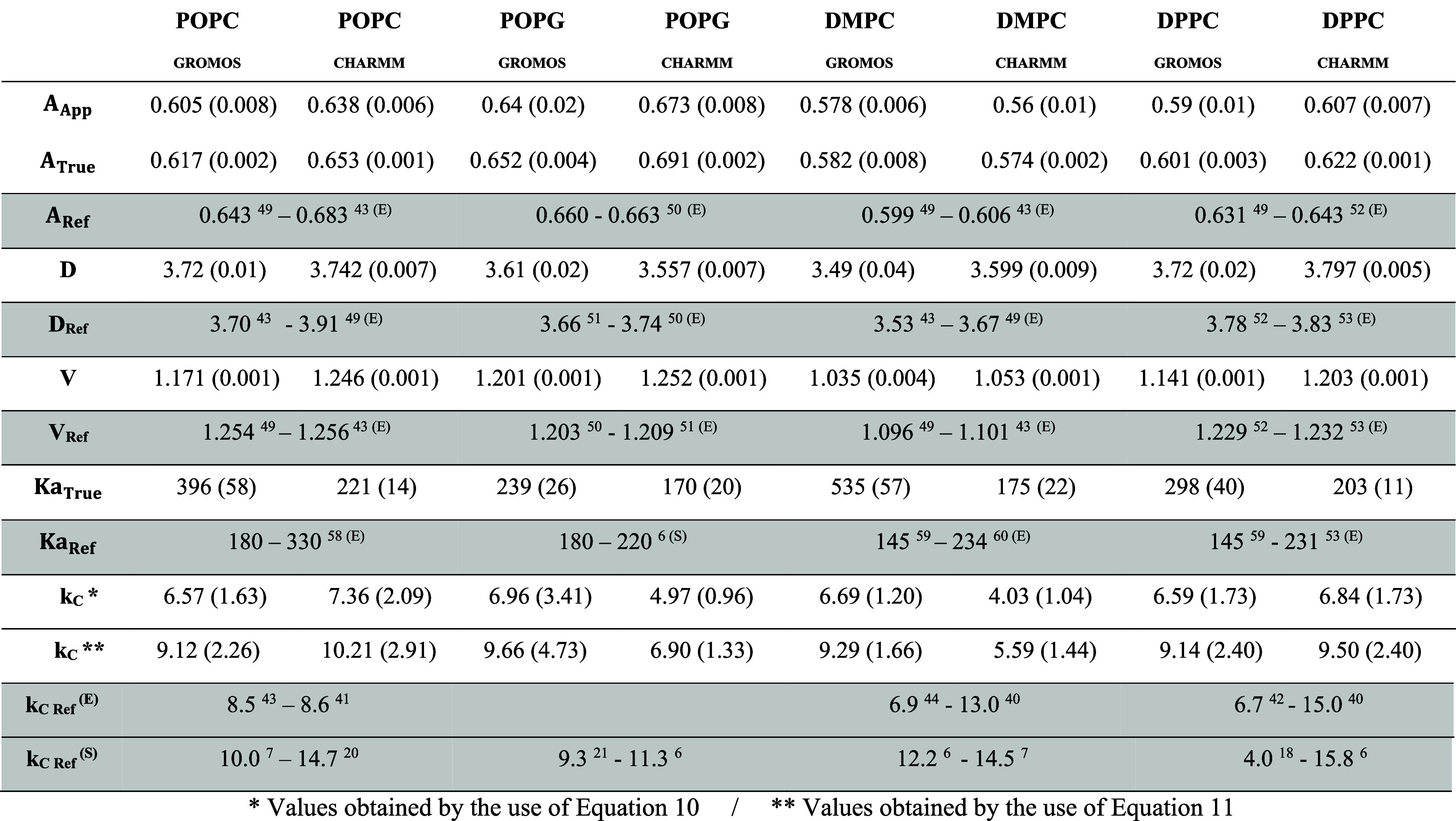
Comparison of Structural and Mechanical Properties from Simulations and Experimental Data^a^

a Quantities are given as area per molecule *A* [nm^2^], bilayer thickness *D* [nm], volume per molecule *V* [nm^3^], compressibility modulus *K_A_* [mN/m], and bending modulus *k*_c_ [10^–20^ J]. References labeled as (E) correspond to experimental data, while those labeled as (S) refer to computational simulations.

## Results

### Structural Analyzes of Simulated Systems

Once simulation and analysis parameters are defined, SuAVE enables direct comparison of membrane structural properties with experimental and computational literature data, as outlined in Figure 1 and Table 1. In general, all geometrical properties for the first replica express statistical convergence for the last 300 ns of the simulations with standard deviations being lower than 2% of the average values estimated (Figure 1). In addition, the probability density function of all properties converges to a Gaussian profile, in accordance with the Central Limit Theorem.^48^ The convergence of the simulations was further assessed by monitoring the skewness of the sampled distributions. Upon convergence, higher-order statistical moments become stationary, notably, skewness approaches zero, indicating symmetric fluctuations around the mean and adequate phase space sampling. Figures SI-3 and SI-4 (Supporting Information) present the corresponding results for replicas 2 and 3.

Comparison of the main structural properties of the simulated bilayers obtained with the GROMOS 54A7 and CHARMM36 force fields is presented in Figure 1. For all four lipid types, both force fields yield values generally consistent with experimental ranges from the literature. Although some differences are observed for specific lipid–force-field combinations, these deviations are not systematic across all systems. These analyses support the conclusion that both force fields provide a comparable description of the membrane structural properties considered in this work. A similar conclusion is supported by the comparison of experimental data with the simulation results (Figure 2 and Table 1). The calculated values show good agreement with experimental results and do not exhibit any systematic dependence on the choice of force field (Table 1). Moreover, relative to previous computational studies reported in the literature,^6,17,19^ the present results are consistent both with the average values summarized in Table 1 and with the expected variability arising from the use of different force fields.

**Figure 2:**
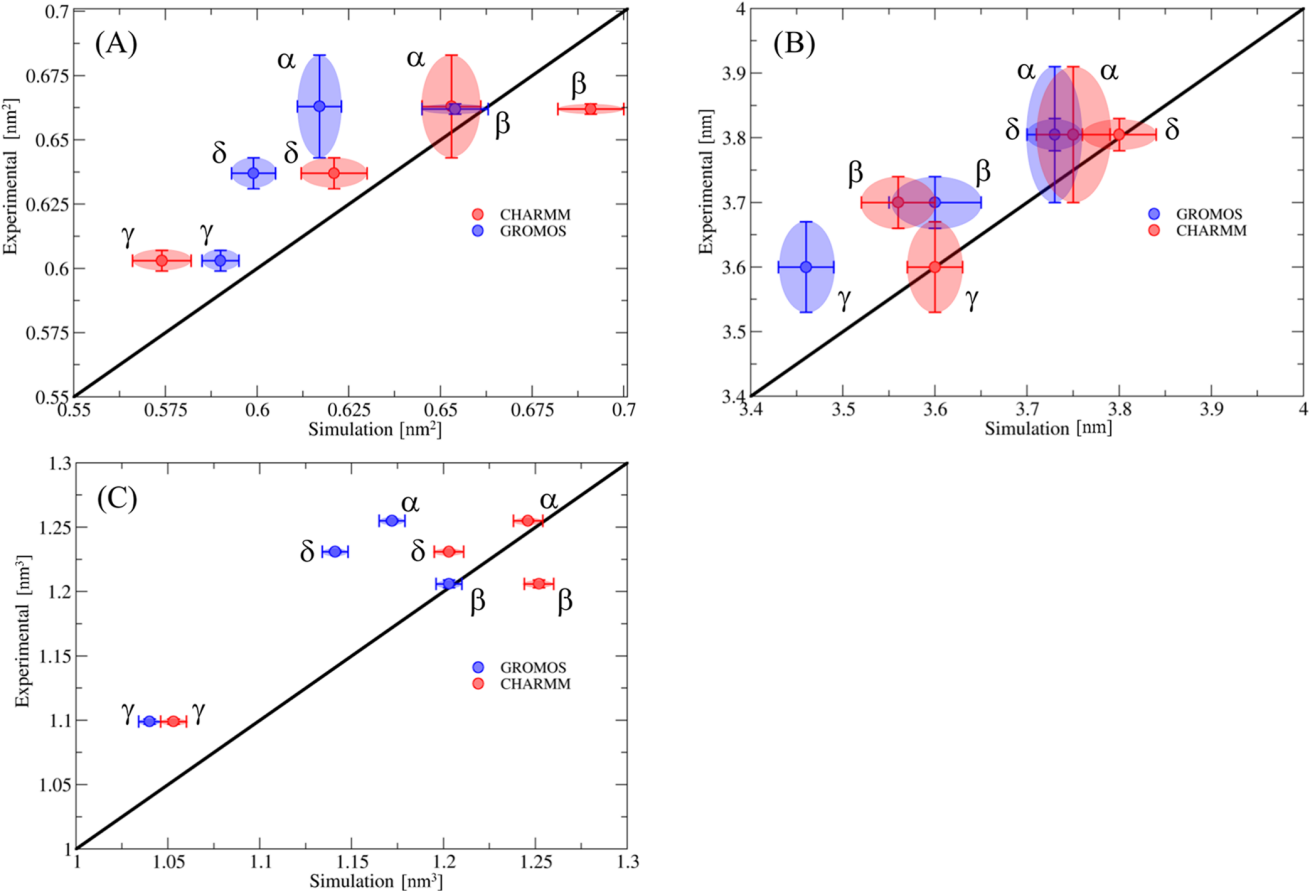
Comparison between experimental data and values obtained from molecular simulations. The experimental error bars (vertical axis) reflect the variability of values reported across different studies in the literature, while the computational error bars (horizontal axis) represent the standard deviations across the independent replicas generated in the simulations performed in this work. The area per lipid is presented in panel A, whereas the membrane thickness and volume per lipid are shown in panels B and C, respectively.^43,49–53^ POPC, POPG, DMPC and DPPC are represented by α, β, γ and δ symbols.

In addition to these results, the curvature order parameter *S*_c_ is able to describe the flatness of the surface as defined by the SuAVE methodology (Figure 1). All profiles yield average values approaching 0.90, confirming that the membrane models remain predominantly flat with limited curvature. These results validate the applicability of the Helfrich framework for describing the membrane Hamiltonian in the simulated systems. Since only thermal fluctuations contributes to the small curvatures captured in the structural properties, the interpretation of fluctuation-derived properties requires consistency with the underlying statistical ensemble, particularly with respect to the correct reproduction of NPT fluctuations.

Within this context, it is important to clarify the scope of the present work, which is a method-validation study rather than a force-field benchmarking effort. The Berendsen pressure coupling does not reproduce the correct NPT fluctuation distribution and may therefore affect fluctuation-derived quantities such as the area compressibility modulus, *K_A_*.^54,55^ In the present work, however, the main objective is to validate the numerical implementation of the SuAVE-based analysis by testing it on trajectories generated with two distinct and well-established atomistic force fields, each employed with its standard simulation protocol, so that the conclusions are not tied to a single parametrization or setup. Accordingly, although the choice of barostat is consistent with the original GROMOS parametrization procedure, it may have resulted in less optimal performance for the current analyses, which rely on the estimation of fluctuation-based properties such as *K_A_*. To address this potential limitation, we performed GROMOS simulations with the Parrinello–Rahman barostat across all four lipid systems to isolate the contributions of force-field parametrization and pressure-coupling scheme (Table SI-7). However, our results should not be interpreted as supporting a general recommendation of one lipid force field over another.

In light of these considerations, once the models have been compared with experimental data and shown to be appropriate for the simulated systems, the methodology employed to calculate their mechanical properties can be systematically evaluated. In other words, we can estimate the values of area compressibility (Ka_true_ e Ka_app_) and bending modulus, *k*_c_. Therefore, by applying eq 4, one can derive the compressibility values corresponding to each area measurement reported in Table 1. However, this estimation requires taking statistical samples from the simulation trajectory to properly capture the variability of compressibility over time. Accordingly, the compressibility values are computed as running averages, with samples systematically collected along the simulation time. This analysis has proven to be informative, highlighting the importance of the statistical treatment of the trajectory and the trajectory length required for the compressibility values to converge toward the experimental results.

The average values obtained from the simulations can deviate substantially from the experimental measurements when fewer than 0.5 × 10^5^ frames from the trajectory are used (Figure 3). However, when more than 1.0 × 10^5^ frames are considered, the average values converge toward the experimental estimates, and the standard deviation becomes negligible. Based on this observation, the compressibility data reported in Table 1 were calculated using the last 300 ns of each simulation, with sets of 2.5 × 10^5^ frames from the trajectory for area compressibility estimates and 1.0 × 10^5^ frames for bending modulus calculation. These sets have also been used for uncertainty assessment of the mechanical properties through the Moving Block Bootstrap protocol. The variability in area compressibility measurements throughout the simulation reflects the inherent sensitivity of this parameter to fluctuations in the area per lipid (Figure 4). Despite achieving statistical convergence of structural properties during the final 300 ns of each simulation (standard deviations <2% of corresponding mean values), small fluctuations in the area per lipid standard deviation produce substantial variations in calculated area compressibility. This behavior follows directly from the mathematical relationship in which area compressibility is inversely proportional to the square of the standard deviation (eq 4). Consequently, modest variations of approximately 5% in the area per lipid standard deviation are sufficient to generate significant changes in computed area compressibility (Figure 4).

**Figure 3:**
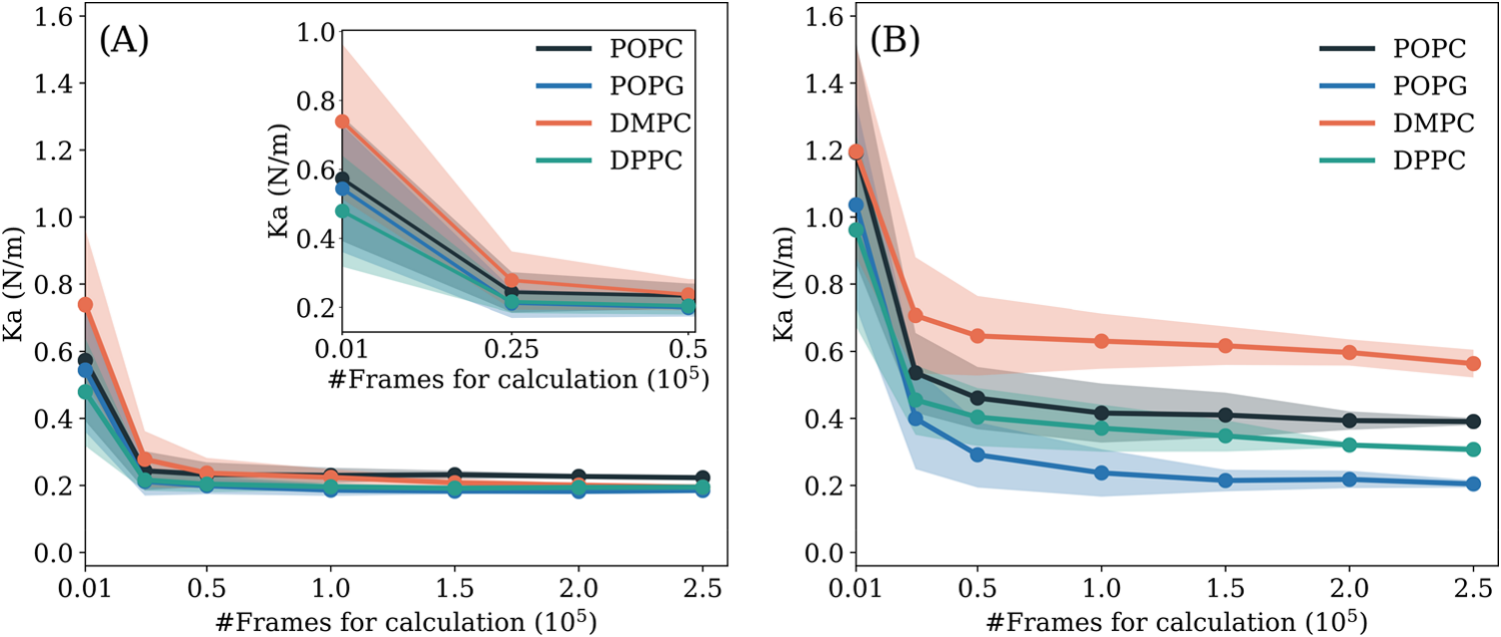
Number of frames required to reach statistical convergence of the area compressibility modulus along the simulation trajectory. Data correspond to replica 1, with frames sampled every 1 ps over the final 300 ns of the simulation. CHARMM results are shown in panel A, whereas GROMOS results are presented in panel B.

**Figure 4:**
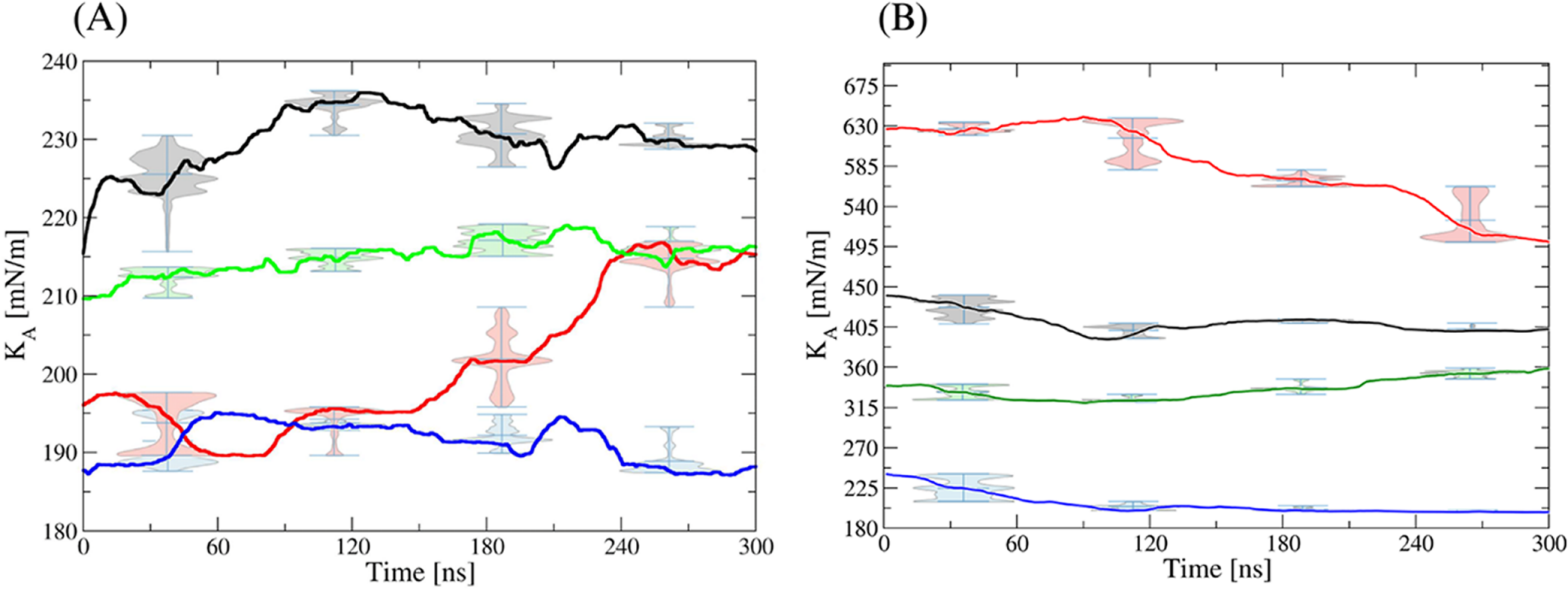
Average of area compressibility for sets of 2.5 × 10^5^ frames taken over the last 300 ns of the trajectory. POPC is represented in black, while POPG in blue, DMPC in red and DPPC in green. CHARMM results are shown in panel A, whereas GROMOS results are presented in panel B.

Furthermore, the intrinsic variability of bending rigidity values calculated from eqs 10 and 11 necessitates appropriate statistical treatment of the bending modulus *k*_c_ (Figure 4). This variability reflects the sensitivity of the bending modulus to fluctuations in the computed parameters. Accordingly, outlier detection was carried out employing the interquartile range (IQR) method,^56,57^ whereby any data point lying below *Q*_1_ – 1.5 × IQR or above *Q*_3_ + 1.5 × IQR was excluded from the bending modulus analysis. Here, *Q*_1_ corresponds to the first quartile (25th percentile) of the distribution, and the IQR is defined as the difference between the third quartile (*Q*_3_) and the first quartile (*Q*_1_). It is important to note that the interquartile range (IQR) filter was employed as a robust statistical criterion to identify extreme values that deviate significantly from the central distribution of the sampled configurations. In this regard, the use of the IQR is not intended to arbitrarily truncate physically meaningful fluctuations, but rather to mitigate the impact of rare, nonrepresentative configurations that may arise from transient numerical instabilities, incomplete equilibration, or sampling artifacts. To assess the impact of the IQR filter, a sensitivity analysis was performed to evaluate the necessity of its application (see Table SI-6 and Figure SI-5). The statistically treated results for the bending modulus *k*_*c*_ obtained from our simulations are summarized in Table 1, together with reference values reported in the literature.

The finite lateral size of the simulated membrane bilayers may influence the estimated elastic properties and warrants explicit consideration. We analyzed 384-lipid systems selected for direct comparison with established literature benchmarks. At this scale, average structural observables converge well, and geometric quantities computed by SuAVE remain stable over the final 300 ns of the trajectories. However, the estimation of mechanical properties such as *K_A_* and particularly *k*_c_ remains subject to the general finite-size limitations of atomistic membrane simulations because the spectrum of long-wavelength fluctuations is restricted by the simulation box and periodic boundary conditions. The SuAVE implementation alleviates one important limitation of Fourier-based area integration, namely the need to define system- and composition-dependent upper integration limits, but it does not make the elastic constants strictly independent of system size. In practical terms, larger membranes would allow better sampling of long-wavelength undulations and may improve the robustness of the mechanical-property estimates, albeit at a substantially increased computational cost and with correspondingly longer simulation times required for convergence. The present results should therefore be interpreted as validation of the proposed methodology for standard atomistic bilayer sizes, rather than as a demonstration of complete finite-size invariance.

Comparison with literature values from both experiments and previous computational studies from,^17^ Lindahl and Edholm,^18^ and Venable, Brown, and Pastor^6^ demonstrates that the calculated structural and mechanical properties are of the expected order of magnitude and in good agreement across all systems analyzed (Table 1). However, GROMOS-based simulations overestimate area compressibility, yielding values approximately twice those observed experimentally^53,58–60^ (Table 1), consistent with findings by Pluhackova et al.^19^ for the same lipid systems. This discrepancy reflects the sensitivity of area compressibility to the choice of intermolecular potentials and force-field parameters. However, the bending modulus values do not exhibit large variations when comparing different parameter sets (Table 1). This behavior can be explained by the fact that the bending modulus is obtained from the difference between the true and projected area compressibilities. Since both quantities are similarly affected by intermolecular potentials, the resulting overestimation largely cancels out, rendering its impact on the bending rigidity negligible. We performed GROMOS simulations with the Parrinello–Rahman barostat across all four lipid systems to isolate the contributions of force-field parametrization and pressure-coupling scheme. Table SI-7 presents the resulting structural and mechanical properties.

The comparison of bending modulus values show that those obtained from eq 10 are systematically lower than those derived from eq 11 (Table 1). Consequently, estimates from eq 10 tend to lie at or below the lower bound of the experimentally reported range, even when they approach experimental values. In contrast, the values obtained from eq 11 are more suitable for comparison with the reference data. However, no clear trend is observed regarding a better agreement with the reference data across different lipid membranes. This behavior may be attributed to the broad range of values reported in the literature, the complexity of experimental measurements, and the diversity of techniques employed.^6,7,18,20,21,40–44^ In fact, as also highlighted by Drabik et al.,^61^ different experimental techniques can yield inconsistent values for the bending rigidity coefficient (*k*_c_), reflecting the sensitivity of this property to both methodological and thermodynamic factors. Likewise, multiple factors beyond temperature, including system size, force field choice, pressure coupling scheme, and equilibration state, significantly influence the mechanical properties of lipid membranes in molecular dynamics simulations.

Therefore, while the values obtained in this work fall within part of the experimental range, it is important to recognize that properties such as *k*_c_ are highly sensitive to temperature variations and to the specific methodology employed. In addition, the results described by Drabik et al.^61^ indicate that membrane morphology can also play a relevant role in the observed values. In particular, measurements performed on vesicles and planar membranes may lead to significantly different estimates of mechanical properties, further contributing to the dispersion reported in the literature. This is readily exemplified in the case of DMPC vesicle with a bending rigidity of 7.22 × 10^–20^ J, while the same lipid membrane in a flat-patch configuration presents a bending rigidity of 11.70 × 10^–20^ J.

### Impact of Membrane Structural Phase on Bending Modulus

Beyond the previously discussed dependence of bending modulus on membrane morphology, the lipid structural phase also represents a key factor in the calculation of the bending modulus within the proposed geometrical and numerical framework.^61^ This dependence is readily understood, since the bending modulus, as described in eqs 10 and 11, depends on the area compressibility which, in turn, is directly influenced by surface fluctuations. This means that membranes ranging from its ordered, solid-like gel phase (Lβ) to a fluid, liquid-crystalline phase (Lα) will present different bending modulus as can be seen in Figure 5.

**Figure 5:**
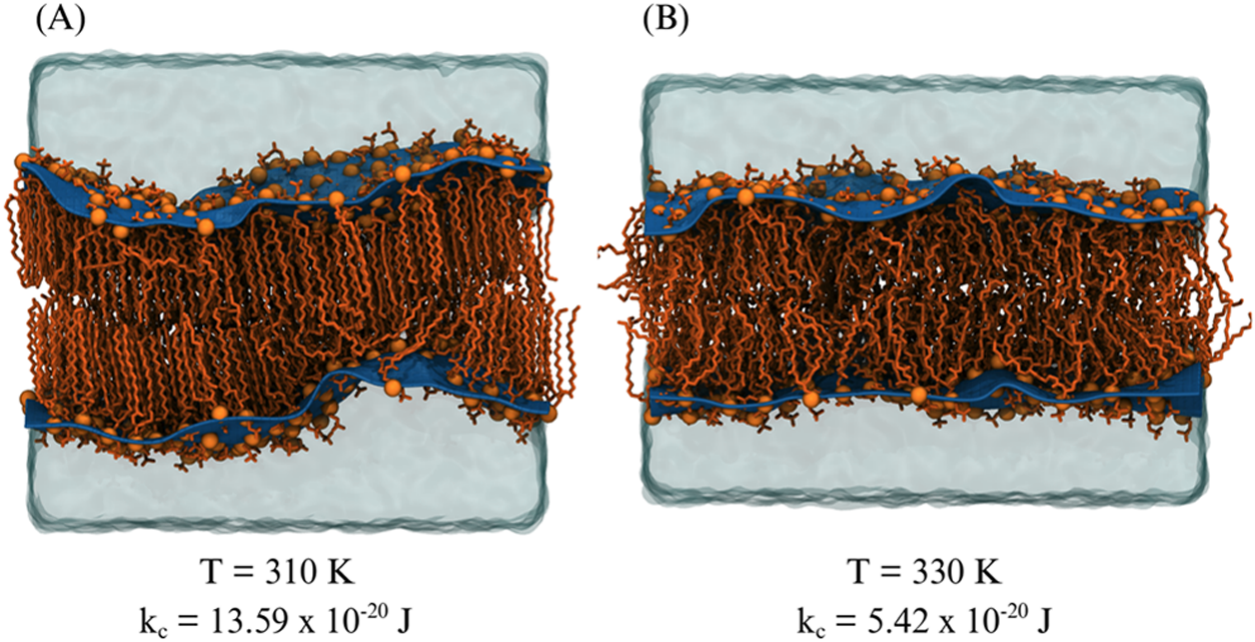
Comparison of the bending modulus calculated for DPPC lipid membrane in (A) an ordered, solid-like gel phase (Lβ) transitioning to ripple phase (Pβ) and (B) in a fluid, liquid-crystalline phase (Lα). Structures and values extracted from CHARMM simulation. Lipids are shown in orange, with phosphate groups highlighted as spheres, while the SuAVE fitted surfaces are depicted in blue. The solvent is represented as a transparent cyan continuum surface.

However, experimental values found on literature are reported above transition temperature, *T*_m_, meaning that simulations must be carried out in a fluid, liquid-crystalline phase (Lα). As mentioned in the Methodology section, in order to satisfy this condition, after equilibration step, simulations of POPC, POPG, and DMPC were conducted at 310 K, whereas the DPPC system was simulated at 330 K. This approach ensures that all data were collected above *T*_m_, therefore, enabling reliable comparison with experimental results. Further discussion of the influence of membrane structural phase on the area compressibility measurements is provided in the Supporting Information.

## Conclusion

Computational simulations of lipid membranes and their elastic properties have been extensively investigated over the past decade, creating a demand for robust computational tools capable of extracting mechanical properties from molecular dynamics trajectories. Among the methodologies developed for this purpose, real-space fluctuation analyses and density-correlation-based approaches have emerged as efficient alternatives for estimating membrane elastic properties. Density-correlation approaches^16^ are based on spatial correlations of membrane density profiles and typically require a smooth, quasi-planar membrane geometry, which restricts their application to highly curved or structurally heterogeneous membranes. Real-space fluctuation methods^13–15^ determine elastic parameters from fluctuations of interfacial atomic positions, operating directly on the probability distribution of local tilt and splay angles of membrane surface. While theoretically transparent, results may be sensitive to the discretization scheme used to represent the surface and contributions from different length scales, ranging from long-wavelength collective undulations to short-wavelength lipid protrusion modes, cannot be separated. The surface integration framework of SuAVE addresses the discretization dependence inherent to real-space approaches and eliminates the need for integration limits defined by lipid molecular dimensions, naturally extending the applicability of the method to arbitrary membrane geometries.

The formalism described by Waheed and Edholm^17^ has been implemented as the *s_comp* tool within the SuAVE software,^38,39^ extending the formalism to arbitrary membrane geometries through the surface integration framework already available in the package. The performance of the proposed methodology was assessed through MD simulations of four distinct phospholipid membranes using two well-established membrane force-fields, namely GROMOS and CHARMM. We demonstrate that the methodology is reliable for well-equilibrated membranes in the fluid liquid-crystalline phase (Lα), for which fluctuation-derived properties are adequately sampled. In gel (Lβ) or ripple (Pβ′) phases, bending fluctuations are strongly suppressed and the isotropic elastic model assumed in the derivation of those equations may not hold, requiring additional caution and validation before the method is applied. It is worth emphasizing that both experimental and computational determinations of membrane elastic constants are technique- and condition-dependent, and substantial variability across studies is commonly observed even under nominally similar thermodynamic conditions (Table 1). Nevertheless, the bending modulus values obtained in the present work are consistent with experimental estimates, particularly those from X-ray scattering, micropipette aspiration, and neutron spin echo experiments, supporting the validity of the proposed approach.

## Supplementary Material



## Data Availability

The structures, topologies, and inputs files are freely available for download at GitHub: https://github.com/SuAVE-Software/Input-files-for-lipid-membrane-simulations.

## Notes

Chemical composition for all simulated systems; relaxation times for structural and mechanical properties; structural and mechanical properties computed for individual replicas in GROMOS and CHARMM simulations; block bootstrap confidence intervals; sensitivity analysis for assessment of bending rigidity with and without IQR filter; benchmarking procedure on selected geometric properties of interest; computed across grids of varying resolutions for the assessment of SuAVE parameters; detailed discussion on the influence of membrane structural phase on the area compressibility measurements and reliability of the mechanical property estimates; structural properties computed for additional replicas; sensitivity analysis for assessment of bending for DPPC in CHARMM simulation (PDF)
