# Supplementary material for: A Numerical Implementation to Calculate Elastic Properties of Biological Membrane Simulations
Source: J Chem Inf Model. 2026 Jul 9;66(14):8203–14. doi: 10.1021/acs.jcim.6c00143 (PMC13418172; doi:10.1021/acs.jcim.6c00143)
Supplement: Supplementary file 1 [file ci6c00143_si_001.pdf]

# SUPPORTING INFORMATION

# A Numerical Implementation to Calculate Elastic Properties of Biological Membrane Simulations

*Denys E. S. Santos[1,*], Vinicius Firmino[2], Ricardo L. Longo[1], Thereza A. Soares[2,3]*

[1]Department of Fundamental Chemistry, Federal University of Pernambuco, 50740-560, Cidade Universitária, Recife, Brazil

[2]Department of Chemistry, FFCLRP, University of São Paulo, 14040-901, Ribeirão Preto, Brazil

[3]Hylleraas Centre for Quantum Molecular Sciences, University of Oslo, 0315, Oslo, Norway

*e-mail: denys.ewerton@ufpe.br

## CHEMICAL COMPOSITION FOR ALL SIMULATED SYSTEMS

**Table SI-1.** Chemical composition of the simulated systems used for data acquisition.

| Force Field | Lipid | lipid:water mass ratio | lipid:water molar ratio | Number of waters | Number of $Na^+$ ions |
|---|---|---|---|---|---|
| GROMOS | POPC | 1.1128 | 0.0264 | 14572.00 | 0.00 |
| | POPG | 0.6157 | 0.0148 | 25952.00 | 384.00 |
| | DMPC | 1.1125 | 0.0295 | 13000.00 | 0.00 |
| | DPPC | 1.4215 | 0.0349 | 11016.00 | 0.00 |
| CHARMM | POPC | 0.8884 | 0.0210 | 18252.00 | 0.00 |
| | POPG | 0.7444 | 0.0179 | 21464.00 | 384.00 |
| | DMPC | 1.1159 | 0.0296 | 12960.00 | 0.00 |
| | DPPC | 0.8908 | 0.0218 | 17579.00 | 0.00 |

## RELAXATION TIMES FOR STRUCTURAL AND MECHANICAL PROPERTIES

**Table SI-2.** Relaxation times (ns) for area per lipid ($A_L$), bilayer thickness ($D$), volume per lipid ($V_L$), curvature order parameter ($S_C$), and area compressibility modulus ($K_A$), reported for each replica and force field.

| | ***Repl 1*** | | ***Repl 2*** | | ***Repl 3*** | |
|---|---|---|---|---|---|---|
| | ***CHARMM*** | ***GROMOS*** | ***CHARMM*** | ***GROMOS*** | ***CHARMM*** | ***GROMOS*** |
| | | | DMPC | | | |
| $\mathbf{A_L}$ | 3.9932 | 7.4307 | 6.4665 | 2.8959 | 1.8300 | 5.7104 |
| **D** | 3.4270 | 12.9940 | 6.4692 | 4.3258 | 2.0182 | 9.0283 |
| $\mathbf{V_L}$ | 2.0540 | 0.5040 | 2.7532 | 1.3343 | 0.8669 | 0.3604 |
| $\mathbf{S_c}$ | 1.8332 | 3.2691 | 1.8271 | 5.2453 | 0.9651 | 12.1823 |
| $\mathbf{K_A}$ | 9.1938 | 9.5201 | 4.3877 | 7.9825 | 6.7693 | 6.5975 |
| | | | DPPC | | | |
| $\mathbf{A_L}$ | 1.1671 | 9.7561 | 1.2370 | 5.0140 | 0.9187 | 5.6609 |
| **D** | 1.3058 | 5.9854 | 1.3549 | 6.2871 | 0.9424 | 7.4220 |
| $\mathbf{V_L}$ | 0.5186 | 2.4150 | 0.4105 | 0.8810 | 0.4105 | 0.7733 |
| $\mathbf{S_c}$ | 0.8666 | 2.9615 | 0.5677 | 1.3149 | 0.7591 | 1.9635 |
| $\mathbf{K_A}$ | 7.2230 | 8.3072 | 1.6915 | 12.7083 | 6.1600 | 6.8405 |
| | | | POPC | | | |
| $\mathbf{A_L}$ | 0.9316 | 4.1044 | 1.2074 | 4.2464 | 2.8882 | 10.8210 |
| **D** | 1.0617 | 6.1768 | 1.3622 | 3.0176 | 1.1734 | 14.0700 |
| $\mathbf{V_L}$ | 0.2438 | 0.8597 | 0.3450 | 1.3597 | 0.3588 | 0.5561 |
| $\mathbf{S_c}$ | 0.6602 | 4.5291 | 0.4230 | 2.1741 | 0.8658 | 7.5957 |
| $\mathbf{K_A}$ | 5.0564 | 4.4322 | 4.8659 | 2.6869 | 8.5592 | 5.9679 |
| | | | POPG | | | |
| $\mathbf{A_L}$ | 1.4958 | 8.8657 | 1.4825 | 9.0428 | 2.8012 | 5.6609 |
| **D** | 1.7319 | 8.8574 | 1.5898 | 9.8246 | 3.1599 | 7.4220 |
| $\mathbf{V_L}$ | 0.3138 | 2.2149 | 0.4954 | 0.4234 | 0.5163 | 0.7733 |
| $\mathbf{S_c}$ | 0.9835 | 2.4761 | 0.8937 | 7.2334 | 0.7898 | 1.9635 |
| $\mathbf{K_A}$ | 4.2287 | 5.9901 | 2.0195 | 4.3405 | 6.1025 | 6.8405 |

## STRUCTURAL AND MECHANICAL PROPERTIES COMPUTED FOR INDIVIDUAL REPLICAS WITH THE GROMOS SIMULATIONS

**Table SI-3.** Structural and mechanical properties from GROMOS simulations. Quantities are given as area per molecule $A_L$ [$nm^2$], bilayer thickness D [nm], volume per molecule $V_L$ [$nm^3$], compressibility modulus $K_A$ [mN/m], bending modulus kc [$10^{-20}$ J] and curvature order parameter $S_C$.

| | ***Repl 1*** | | ***Repl 2*** | | ***Repl 3*** | |
|---|---|---|---|---|---|---|
| | *<X>* | $\sigma_X$ | *<X>* | $\sigma_X$ | *<X>* | $\sigma_X$ |
| | | | DMPC | | | |
| **$A_L$** | 0.5902 | 0.0006 | 0.5809 | 0.0006 | 0.5753 | 0.0007 |
| **D** | 3.4548 | 0.0037 | 3.4875 | 0.0037 | 3.5259 | 0.0042 |
| **$V_L$** | 1.0392 | 0.0003 | 1.0324 | 0.0005 | 1.0335 | 0.0003 |
| **$S_c$** | 0.9043 | 0.0013 | 0.8993 | 0.0017 | 0.8937 | 0.0020 |
| **$K_A$** | 593.8200 | 12.1190 | 484.0200 | 5.4275 | 527.9500 | 6.3447 |
| **$k_C$*** | 7.1941 | 0.6234 | 5.9286 | 0.4003 | 6.9550 | 0.6605 |
| **$k_C$**** | 9.9885 | 0.8656 | 8.2314 | 0.5558 | 9.6565 | 0.9170 |
| | | | DPPC | | | |
| **$A_L$** | 0.5993 | 0.0010 | 0.5988 | 0.0008 | 0.6035 | 0.0012 |
| **D** | 3.7336 | 0.0054 | 3.7406 | 0.0042 | 3.7113 | 0.0061 |
| **$V_L$** | 1.1404 | 0.0006 | 1.1416 | 0.0005 | 1.1414 | 0.0007 |
| **$S_c$** | 0.8978 | 0.0016 | 0.8986 | 0.0010 | 0.8935 | 0.0012 |
| **$K_A$** | 332.6800 | 2.7008 | 290.1300 | 22.9370 | 269.7500 | 0.8851 |
| **$k_C$*** | 5.9179 | 0.4070 | 5.6572 | 0.5026 | 8.1798 | 0.8102 |
| **$k_C$**** | 8.2166 | 0.5651 | 7.8546 | 0.6979 | 11.3570 | 1.1249 |
| | | | POPC | | | |
| **$A_L$** | 0.6173 | 0.0009 | 0.6161 | 0.0007 | 0.6174 | 0.0013 |
| **D** | 3.7239 | 0.0050 | 3.7291 | 0.0032 | 3.7192 | 0.0076 |
| **$V_L$** | 1.1716 | 0.0004 | 1.1709 | 0.0005 | 1.1703 | 0.0004 |
| **$S_c$** | 0.9012 | 0.0015 | 0.9033 | 0.0012 | 0.9057 | 0.0022 |
| **$K_A$** | 408.5700 | 1.7643 | 446.5600 | 0.8200 | 332.2700 | 0.7000 |
| **$k_C$*** | 5.7395 | 0.5526 | 5.6895 | 0.1473 | 8.2785 | 0.3681 |
| **$k_C$**** | 7.9688 | 0.7673 | 7.8994 | 0.2045 | 11.4940 | 0.5111 |
| | | | POPG | | | |
| **$A_L$** | 0.6536 | 0.0018 | 0.6490 | 0.0013 | 0.6543 | 0.0012 |
| **D** | 3.6081 | 0.0075 | 3.6287 | 0.0066 | 3.6030 | 0.0065 |
| **$V_L$** | 1.2020 | 0.0006 | 1.2003 | 0.0003 | 1.2014 | 0.0004 |
| **$S_c$** | 0.8957 | 0.0014 | 0.9007 | 0.0019 | 0.8997 | 0.0011 |
| **$K_A$** | 209.1400 | 2.5185 | 253.3400 | 1.0804 | 254.1900 | 2.4733 |
| **$k_C$*** | 6.2920 | 0.9030 | 10.5127 | 0.1737 | 4.0610 | 0.1443 |
| **$k_C$**** | 8.7360 | 1.2537 | 14.5960 | 0.2412 | 5.6384 | 0.2003 |

* Values obtained by the use of Equation 10 / ** Values obtained by the use of Equation 11

## STRUCTURAL AND MECHANICAL PROPERTIES COMPUTED FOR INDIVIDUAL REPLICAS WITH THE CHARMM SIMULATIONS

**Table SI-4.** Structural and mechanical properties from CHARMM simulations. Quantities are given as area per molecule $A_L$ [nm$^2$], bilayer thickness D [nm], volume per molecule $V_L$ [nm$^3$], compressibility modulus $K_A$ [mN/m], bending modulus kc [$10^{-20}$ J] and curvature order parameter $S_C$.

| | ***Repl 1*** | | ***Repl 2*** | | ***Repl 3*** | |
|---|---|---|---|---|---|---|
| | *<X>* | $\sigma_X$ | *<X>* | $\sigma_X$ | *<X>* | $\sigma_X$ |
| | | | DMPC | | | |
| $A_L$ | 0.5742 | 0.0011 | 0.5743 | 0.0017 | 0.5746 | 0.0008 |
| **D** | 3.5988 | 0.0050 | 3.5998 | 0.0069 | 3.5983 | 0.0034 |
| $V_L$ | 1.0530 | 0.0007 | 1.0535 | 0.0009 | 1.0537 | 0.0004 |
| $S_c$ | 0.8851 | 0.0016 | 0.8851 | 0.0016 | 0.8841 | 0.0009 |
| $K_A$ | 197.7800 | 2.4795 | 153.2500 | 0.1058 | 175.3400 | 0.2234 |
| $k_C$* | 4.4230 | 0.4446 | 4.5398 | 0.4944 | 3.1158 | 0.0960 |
| $k_C$** | 6.1410 | 0.6172 | 6.3032 | 0.6865 | 4.3261 | 0.1333 |
| | | | DPPC | | | |
| $A_L$ | 0.6219 | 0.0007 | 0.6215 | 0.0007 | 0.6218 | 0.0006 |
| **D** | 3.7965 | 0.0033 | 3.7982 | 0.0032 | 3.7970 | 0.0027 |
| $V_L$ | 1.2030 | 0.0004 | 1.2029 | 0.0004 | 1.2030 | 0.0003 |
| $S_c$ | 0.8821 | 0.0007 | 0.8814 | 0.0007 | 0.8827 | 0.0006 |
| $K_A$ | 215.2800 | 0.5638 | 196.6100 | 0.2984 | 196.3800 | 1.0017 |
| $k_C$* | 5.2174 | 0.3737 | 7.2065 | 0.8124 | 8.1085 | 0.0870 |
| $k_C$** | 7.2440 | 0.5189 | 10.0057 | 1.1280 | 11.2580 | 0.1208 |
| | | | POPC | | | |
| $A_L$ | 0.6526 | 0.0006 | 0.6539 | 0.0006 | 0.6538 | 0.0010 |
| **D** | 3.7462 | 0.0025 | 3.7407 | 0.0026 | 3.7390 | 0.0042 |
| $V_L$ | 1.2456 | 0.0003 | 1.2466 | 0.0003 | 1.2458 | 0.0005 |
| $S_c$ | 0.8945 | 0.0006 | 0.8953 | 0.0007 | 0.8952 | 0.0011 |
| $K_A$ | 231.6300 | 0.5873 | 228.6900 | 0.9574 | 204.5500 | 0.5682 |
| $k_C$* | 6.4269 | 0.4547 | 6.1444 | 0.8331 | 9.4971 | 0.1418 |
| $k_C$** | 8.9233 | 0.6313 | 8.5310 | 1.1567 | 13.1860 | 0.1969 |
| | | | POPG | | | |
| $A_L$ | 0.6910 | 0.0008 | 0.6902 | 0.0007 | 0.6917 | 0.0011 |
| **D** | 3.5577 | 0.0035 | 3.5582 | 0.0029 | 3.5540 | 0.0046 |
| $V_L$ | 1.2526 | 0.0003 | 1.2513 | 0.0004 | 1.2524 | 0.0004 |
| $S_c$ | 0.8765 | 0.0011 | 0.8783 | 0.0010 | 0.8785 | 0.0013 |
| $K_A$ | 192.4000 | 0.4337 | 163.7700 | 0.4184 | 153.0200 | 1.0475 |
| $k_C$* | 5.9091 | 0.3202 | 4.3842 | 0.1595 | 4.6116 | 0.3351 |
| $k_C$** | 8.2043 | 0.4446 | 6.0871 | 0.2214 | 6.4029 | 0.4653 |

* Values obtained by the use of Equation 10 / ** Values obtained by the use of Equation 11

## BLOCK BOOTSTRAP CONFIDENCE INTERVALS

**Table SI-5.** Block bootstrap confidence intervals for compressibility modulus $K_A$ [mN/m], for each replica and force field.

| ***Repl 1*** | | ***Repl 2*** | | ***Repl 3*** | |
|---|---|---|---|---|---|
| ***CHARMM*** | ***GROMOS*** | ***CHARMM*** | ***GROMOS*** | ***CHARMM*** | ***GROMOS*** |
| DMPC | | | | | |
| 195.59 – 201.25 | 585.03 – 608.81 | 153.18 – 153.32 | 480.20 – 490.35 | 175.20 – 175.40 | 523.35 – 536.13 |
| DPPC | | | | | |
| 214.91 – 215.89 | 330.28 – 335.47 | 196.45 – 196.74 | 273.61 – 298.71 | 195.56 – 197.53 | 268.96 – 270.57 |
| POPC | | | | | |
| 231.28 – 232.20 | 407.14 – 410.22 | 227.89 – 229.54 | 445.97 – 447.05 | 204.13 – 204.96 | 331.65 – 333.13 |
| POPG | | | | | |
| 192.13 – 192.57 | 207.01 – 211.31 | 163.34 – 164.11 | 252.59 – 254.93 | 152.26 – 153.96 | 252.42 – 254.82 |

## SENSITIVITY ANALYSIS FOR ASSESSMENT OF BENDING RIGIDITY WITH AND WITHOUT IQR FILTER

**Table SI-6.** Sensitivity analysis for assessment of bending rigidity, $k_c$ ($10^{-20}$ J) with and without Inter-Quartile Range filter for each replica and force field.

| | ***Repl 1*** | | ***Repl 2*** | | ***Repl 3*** | |
|---|---|---|---|---|---|---|
| | ***CHARMM*** | ***GROMOS*** | ***CHARMM*** | ***GROMOS*** | ***CHARMM*** | ***GROMOS*** |
| DMPC | | | | | | |
| With IQR | 6.1410 (0.618) | 9.9885 (0.866) | 6.3032 (0.887) | 8.2314 (0.556) | 4.3261 (0.134) | 9.6565 (0.917) |
| Without IQR | 8.6166 (1.366) | 13.133 (2.813) | 8.3981 (1.166) | 13.299 (0.832) | 4.7481 (0.496) | 11.4840 (1.503) |
| DPPC | | | | | | |
| With IQR | 7.2440 (0.519) | 8.2166 (0.565) | 10.0057 (1.128) | 7.8546 (0.698) | 11.258 (0.121) | 11.3570 (1.125) |
| Without IQR | 24.237 (1.765) | 9.5733 (0.985) | 15.5230 (3.798) | 11.6136 (2.177) | 17.9091 (3.045) | 26.1517 (0.536) |
| POPC | | | | | | |
| With IQR | 8.9233 (0.631) | 7.9687 (0.768) | 8.531 (1.157) | 7.8994 (0.204) | 13.1860 (0.197) | 11.4940 (0.511) |
| Without IQR | 9.9309 (0.977) | 8.1247 (0.858) | 11.4125 (2.469) | 11.0034 (1.397) | 18.1825 (1.724) | 17.1855 (5.527) |
| POPG | | | | | | |
| With IQR | 8.2043 (0.445) | 8.736 (1.254) | 6.0871 (0.221) | 14.596 (0.241) | 6.4029 (0.465) | 5.6384 (0.200) |
| Without IQR | 9.9771 (0.813) | 13.0588 (0.494) | 8.3159 (1.830) | 20.7573 (1.011) | 7.0931 (0.682) | 7.7278 (1.564) |

## STRUCTURAL AND MECHANICAL PROPERTIES COMPUTED IN GROMOS SIMULATIONS WITH PARRINELLO-RAHMAN BAROSTAT

Control simulations of all four lipid systems were performed using the GROMOS 54A7 force field with the Parrinello-Rahman barostat in place of the Berendsen algorithm to assess the relative contributions of force-field parametrization and pressure-coupling scheme to the mechanical properties reported in the manuscript. Structural and mechanical properties extracted from these trajectories using the SuAVE *s_comp* tool are reported in Table SI-7, alongside the corresponding Berendsen-based values for direct comparison. For three of the four systems (DMPC, POPC, and POPG), the barostat-induced changes in $K_A$ are substantially smaller than those arising from force-field differences between GROMOS and CHARMM, suggesting that the elevated $K_A$ values observed in the GROMOS simulations are primarily of force-field origin rather than a consequence of fluctuation suppression by the Berendsen scheme. For the DPPC system, the Parrinello-Rahman simulation converges to an area per lipid of 0.543 $nm^2$, well below both the Berendsen result (0.599 $nm^2$) and the experimental Lα-phase range, suggesting that this system does not maintain the liquid-crystalline phase under the modified protocol at 330 K, and may not be directly compared with experimental references of Lα-phase DPPC membranes. A systematic investigation of the effect of pressure-coupling schemes on fluctuation-derived membrane properties, including the associated convergence requirements, remains an open methodological question beyond the scope of the present work.

**Table SI-7.** Structural and mechanical properties from GROMOS simulations with different barostat. Quantities are given as area per molecule $A_L$ [nm$^2$], bilayer thickness D [nm], volume per molecule $V_L$ [nm$^3$], compressibility modulus $K_A$ [mN/m], and bending modulus kc [$10^{-20}$ J]. For comparison purposes, the values corresponding to Replica 1 are shown for each barostat configuration, as averages extracted from Moving Block Bootstrap Protocol.

| | ***Berendsen*** | | ***Parrinello-Rahman*** | |
|---|---|---|---|---|
| | <*X*> | $\sigma_X$ | <*X*> | $\sigma_X$ |
| | | **DMPC** | | |
| $\mathbf{A_L}$ | 0.5902 | 0.0006 | 0.5827 | 0.0006 |
| **D** | 3.4548 | 0.0037 | 3.4753 | 0.0034 |
| $\mathbf{V_L}$ | 1.0392 | 0.0003 | 1.0321 | 0.0005 |
| $\mathbf{S_c}$ | 0.9043 | 0.0013 | 0.9010 | 0.0017 |
| $\mathbf{K_A}$ | 593.8200 | 12.1190 | 555.4200 | 6.3390 |
| $\mathbf{k_C}$ * | 7.1941 | 0.6234 | 10.4290 | 1.1614 |
| $\mathbf{k_C}$ ** | 9.9885 | 0.8656 | 14.2990 | 1.6171 |
| | | **DPPC** | | |
| $\mathbf{A_L}$ | 0.5993 | 0.0010 | 0.5434 | 0.0004 |
| **D** | 3.7336 | 0.0054 | 3.9575 | 0.0026 |
| $\mathbf{V_L}$ | 1.1404 | 0.0006 | 1,0943 | 0.0007 |
| $\mathbf{S_c}$ | 0.8978 | 0.0016 | 0.8205 | 0.0019 |
| $\mathbf{K_A}$ | 332.6800 | 2.7008 | 475.6900 | 71.5000 |
| $\mathbf{k_C}$ * | 5.9179 | 0.4070 | 5.9703 | 1.1547 |
| $\mathbf{k_C}$ ** | 8.2166 | 0.5651 | 8.3669 | 1.5615 |
| | | **POPC** | | |
| $\mathbf{A_L}$ | 0.6173 | 0.0009 | 0.6049 | 0.0009 |
| **D** | 3.7239 | 0.0050 | 3.7591 | 0.0051 |
| $\mathbf{V_L}$ | 1.1716 | 0.0004 | 1.1588 | 0.0002 |
| $\mathbf{S_c}$ | 0.9012 | 0.0015 | 0.9112 | 0.0019 |
| $\mathbf{K_A}$ | 408.5700 | 1.7643 | 492.7600 | 16.0630 |
| $\mathbf{k_C}$ * | 5.7395 | 0.5526 | 8.9188 | 2.2698 |
| $\mathbf{k_C}$ ** | 7.9688 | 0.7673 | 12.3200 | 3.0011 |
| | | **POPG** | | |
| $\mathbf{A_L}$ | 0.6536 | 0.0018 | 0.6266 | 0.0017 |
| **D** | 3.6081 | 0.0075 | 3.7202 | 0.0072 |
| $\mathbf{V_L}$ | 1.2020 | 0.0006 | 1.1881 | 0.0007 |
| $\mathbf{S_c}$ | 0.8957 | 0.0014 | 0.8968 | 0.0029 |
| $\mathbf{K_A}$ | 209.1400 | 2.5185 | 276.4300 | 0.4900 |
| $\mathbf{k_C}$ * | 6.2920 | 0.9030 | 7.5724 | 1.4294 |
| $\mathbf{k_C}$ ** | 8.7360 | 1.2537 | 10.4530 | 1.9054 |

*Values obtained by the use of Equation 10; ** Values obtained by the use of Equation 11.

## INFLUENCE OF MEMBRANE STRUCTURAL PHASE ON THE AREA COMPRESSIBILITY MEASUREMENTS

As discussed in the Methodology section, for the simulations to yield reliable mechanical properties that can be meaningfully compared with experimental values, as given by Equations 10 and 11, the bending modulus, the $K_A^{\mathrm{true}}$ must be greater than $K_A^{\mathrm{app}}$, which is the case for the simulated systems above the transition temperature. However, this limitation affects not only the structural phase of the lipid membrane but also the magnitude of area fluctuations, which directly influences the area compressibility calculated using Equation 4. Once on its ordered, solid-like gel phase ($L_\beta$), lipid membranes may exhibit a marked reduction in area fluctuations for the true and projected areas. This behavior may lead to a significant overestimation of the $K_A^{\mathrm{app}}$ relative to $K_A^{\mathrm{true}}$, therefore compromising the reliability of the mechanical property estimates

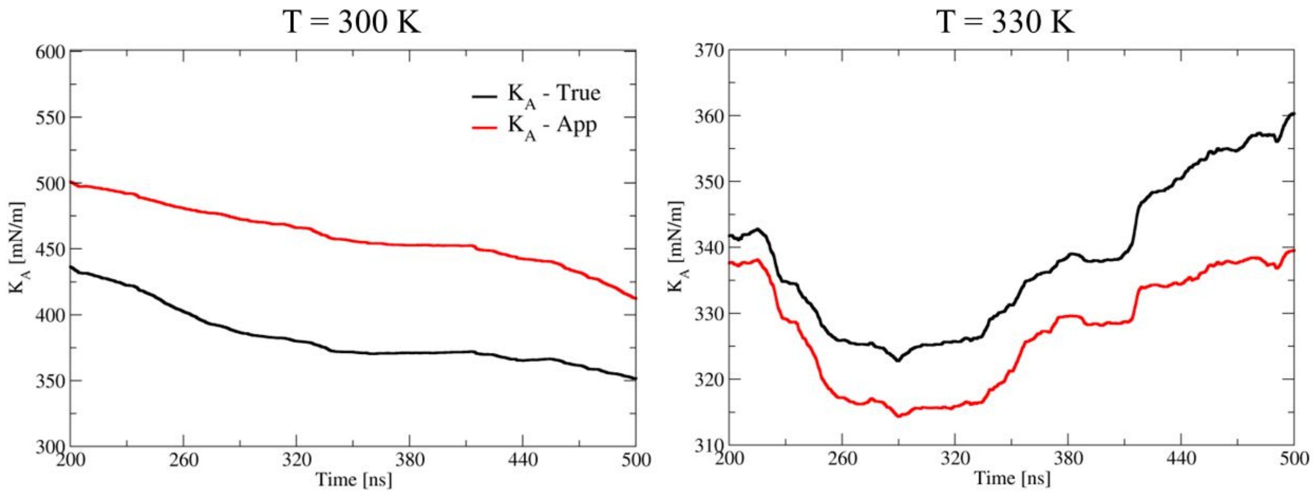


**Figure SI-1.** Area compressibilities calculated for DPPC above and below transition temperature, $T_m$. Values extracted from GROMOS simulation.

As can be seen in Figure SI-1, simulations below $T_m$ may undergo an inversion in the area compressibility values, rendering the bending modulus calculation impossible. Although this is not always observed, it should be taken into consideration when estimating the mechanical properties of lipid membranes, particularly in systems that have not yet reached statistical convergence of structural parameters, such as the area per lipid.

## ASSESSMENT OF SUAVE PARAMETERS

Prior to starting the analysis of the results, it is imperative to undertake an evaluation of the methodology employed in the analysis. It is well-established, based on the development of the algorithm implemented in SuAVE, that the resolution of the fitting grid significantly influences the outcomes of certain geometric properties. This observation intimates that the requisite approximations for accurate measurement may not be entirely met. Consequently, in order to overcome this issue, it is necessary to conduct a benchmarking procedure on selected geometric properties of interest, computed across grids of varying resolutions. In the context of our specific investigation, this benchmarking was exemplified through the computation of the surface area of the system comprising POPC, modeled within the GROMOS force field. The results obtained may be observed in Figure SI-2. As can be seen, a bin resolution of 100 is already adequate for achieving convergence of the property under consideration. Henceforth, it is not necessary to increase the resolution of the fitting grid and all analyses will be conducted using this parameter to ensure comparability of results across different systems.

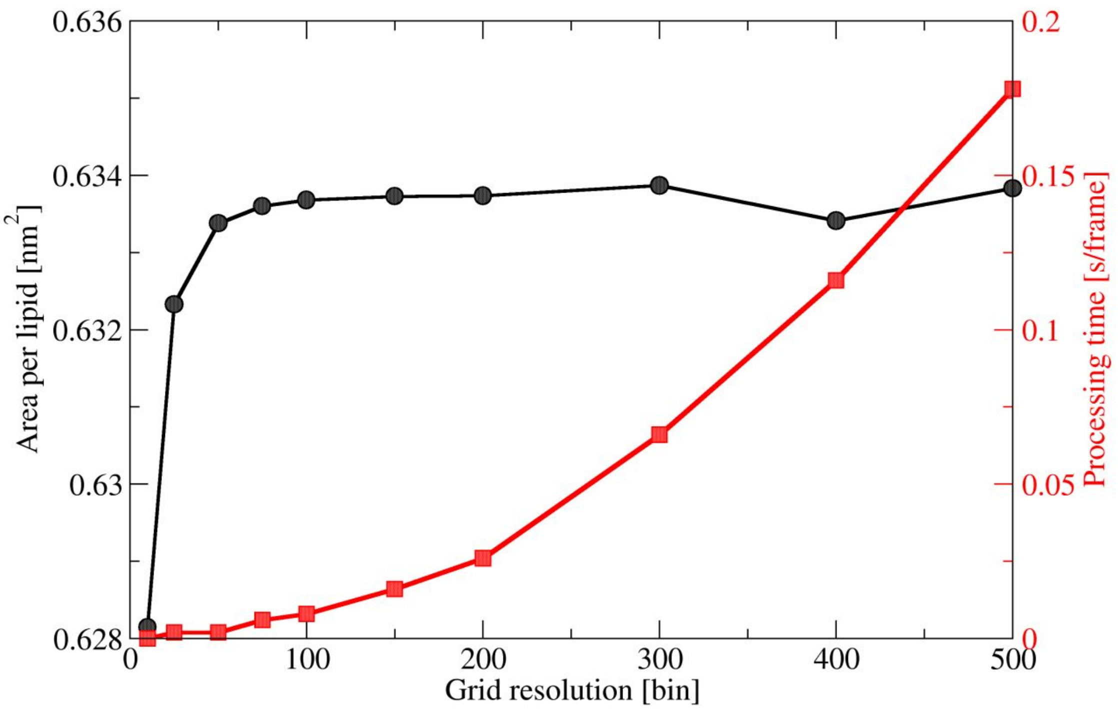


**Figure SI-2.** Assessment of the convergence of the average area per lipid for the first 500 frames of the trajectory of the system composed of POPC simulated with the GROMOS force field and as a function of the resolution of the fitting grid. Average area per lipid (black) and computation time (red) are shown as function of the grid surface resolution.

## STRUCTURAL PROPERTIES COMPUTED FOR ADDITIONAL REPLICAS

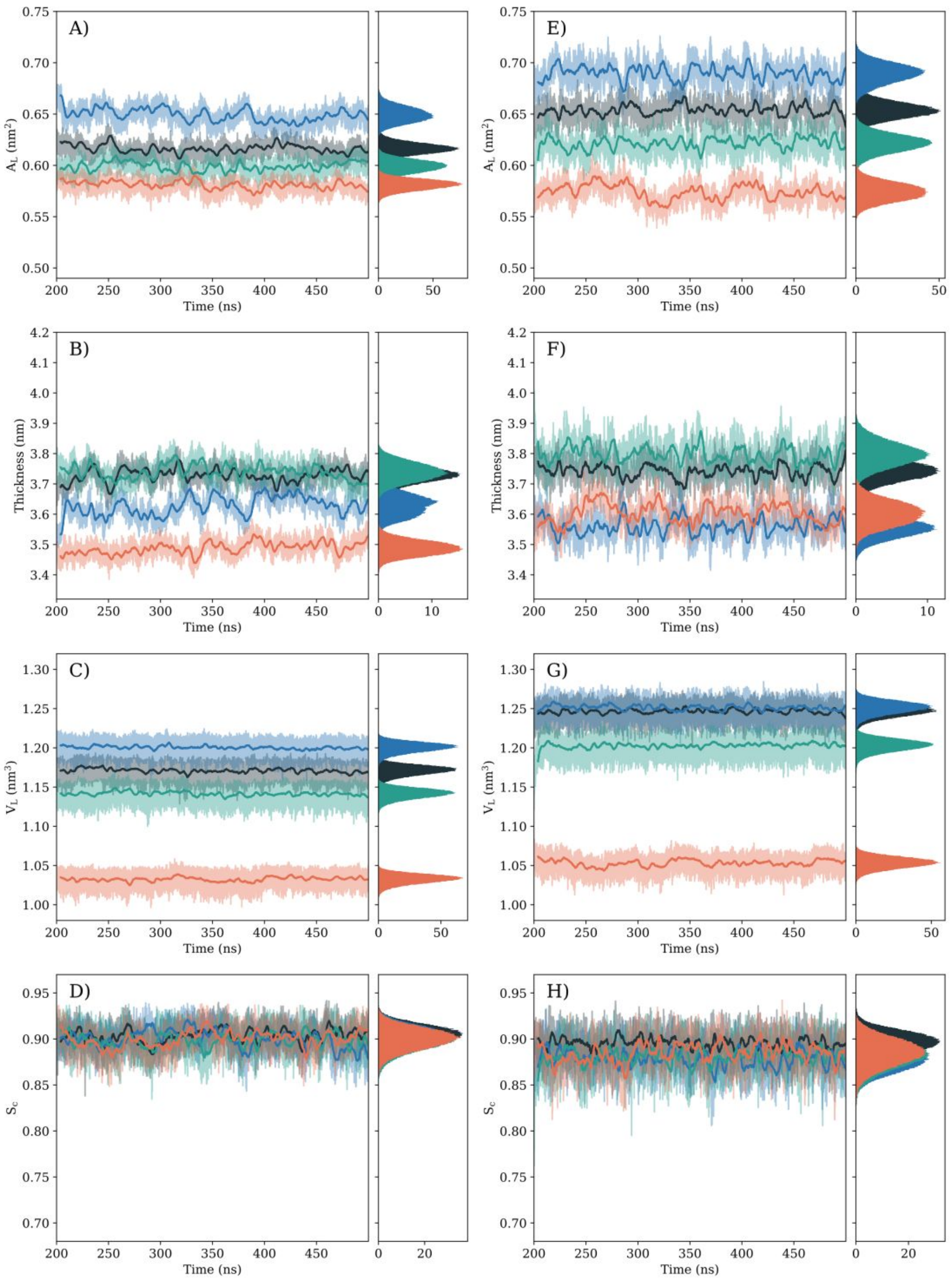


**Figure SI-3.** Assessment of the area per lipid (A and E), thickness (B and F), volume per lipid (C and G) and curvature order parameter (D and H), obtained with SuAVE (bin=100), for replica 2. Trajectory averages of the last 300 ns. Simulations with GROMOS 54A7 are represented at left while simulations with CHARMM 36 at right. POPC is represented in black, while POPG in blue, DMPC in red and DPPC in green.

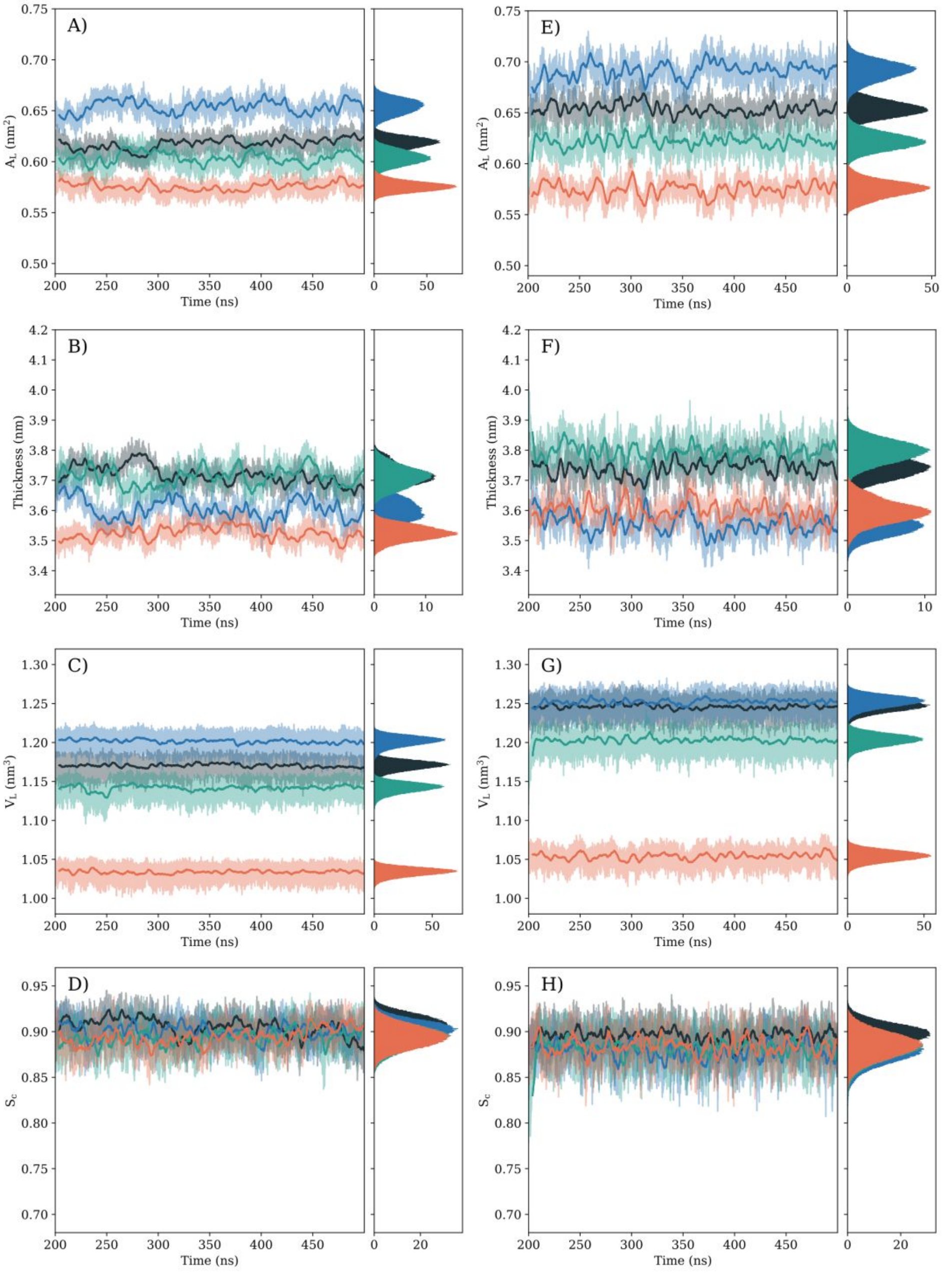


**Figure SI-4.** Assessment of the area per lipid (A and E), thickness (B and F), volume per lipid (C and G) and curvature order parameter (D and H), obtained with SuAVE (bin=100), for replica 3. Trajectory averages of the last 300 ns. Simulations with GROMOS 54A7 are represented at left while simulations with CHARMM 36 at right. POPC is represented in black, while POPG in blue, DMPC in red and DPPC in green.

**SENSITIVITY ANALYSIS FOR ASSESSMENT OF BENDING FOR DPPC IN CHARMM SIMULATION**

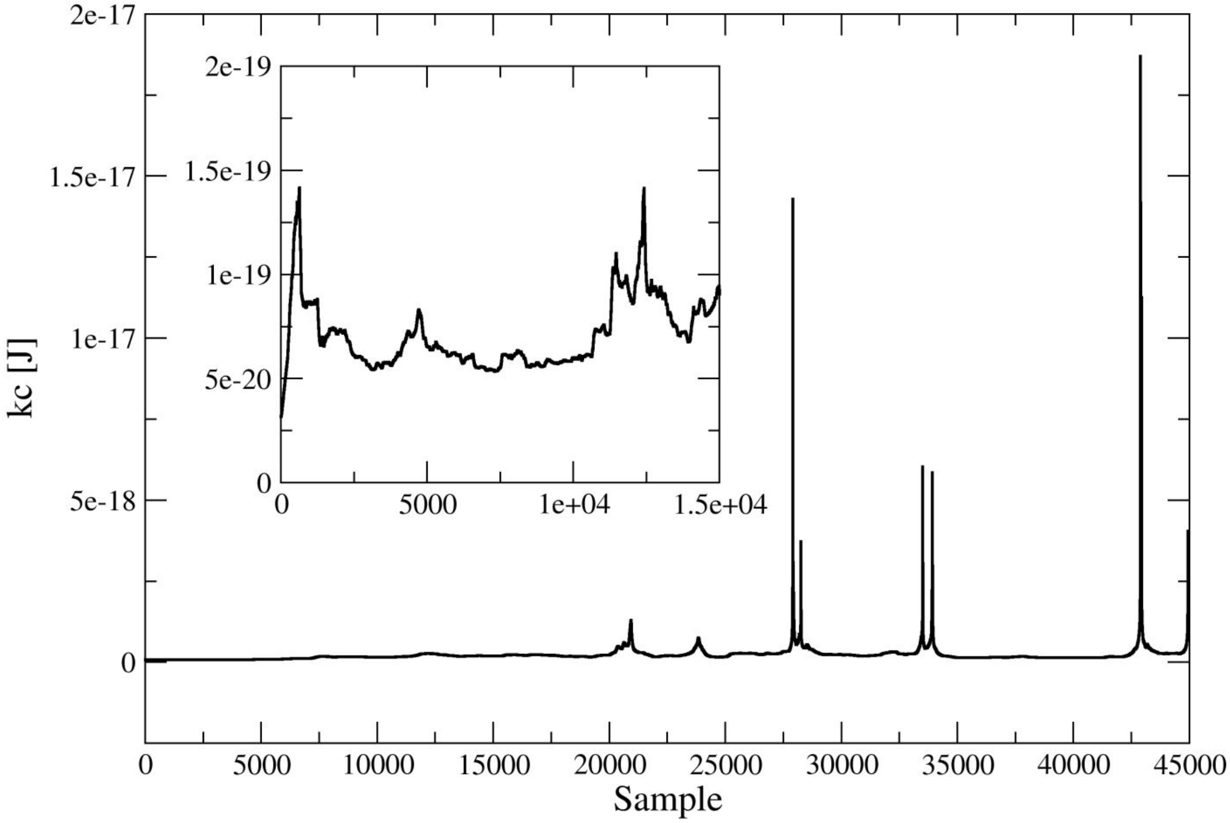


**Figure SI-5**. Bending rigidity calculated for replica 1 of DPPC in CHARMM simulations as a function of the number of samples obtained from area compressibility measurements.
